# Forest biomass density across large climate gradients in northern South America is related to water availability but not with temperature

**DOI:** 10.1371/journal.pone.0171072

**Published:** 2017-03-16

**Authors:** Esteban Álvarez-Dávila, Luis Cayuela, Sebastián González-Caro, Ana M. Aldana, Pablo R. Stevenson, Oliver Phillips, Álvaro Cogollo, Maria C. Peñuela, Patricio von Hildebrand, Eliana Jiménez, Omar Melo, Ana Catalina Londoño-Vega, Irina Mendoza, Oswaldo Velásquez, Fernando Fernández, Marcela Serna, Cesar Velázquez-Rua, Doris Benítez, José M. Rey-Benayas

**Affiliations:** 1 Departamento de Ciencias de la Vida, Universidad de Alcalá, Alcalá, Spain; 2 Grupo de Servicios Ecosistemicos y Cambio Climático, Fundación Convida, Medellín, Colombia; 3 Escuela de Ciencias Agrícolas, Pecuarias y del Medio Ambiente, Universidad Nacional Abierta y a Distancia, Medellín, Colombia; 4 Área de Biodiversidad y Conservación, Escuela Superior de Ciencias Experimentales y Tecnológicas, Universidad Rey Juan Carlos, Departamental I, Madrid, Spain; 5 Herbario JAUM, Jardín Botánico de Medellín, Medellín, Colombia; 6 Departamento de Ciencias Biológicas, Universidad de Los Andes, Bogotá, Colombia; 7 School of Geography, University of Leeds, Leeds, United Kingdom; 8 Universidad Regional Amazónica, Napo, Ecuador; 9 Fundación Puerto Rastrojo, Bogotá, Colombia; 10 Grupo de Ecología de Ecosistemas Terrestres Tropicales, Universidad Nacional de Colombia, Bogotá, Colombia; 11 Facultad de Ingeniería Forestal, Universidad del Tolima, Ibagué, Colombia; 12 Independent investigator, Medellín, Colombia; 13 Facultad de Ciencias Agronómicas, Universidad Nacional de Colombia, Medellín, Colombia; 14 Institución Universitaria Tecnológico de Antioquia, Facultad de Ingeniería, Medellín, Colombia; The Ohio State University, UNITED STATES

## Abstract

Understanding and predicting the likely response of ecosystems to climate change are crucial challenges for ecology and for conservation biology. Nowhere is this challenge greater than in the tropics as these forests store more than half the total atmospheric carbon stock in their biomass. Biomass is determined by the balance between biomass inputs (i.e., growth) and outputs (mortality). We can expect therefore that conditions that favor high growth rates, such as abundant water supply, warmth, and nutrient-rich soils will tend to correlate with high biomass stocks. Our main objective is to describe the patterns of above ground biomass (AGB) stocks across major tropical forests across climatic gradients in Northwestern South America. We gathered data from 200 plots across the region, at elevations ranging between 0 to 3400 m. We estimated AGB based on allometric equations and values for stem density, basal area, and wood density weighted by basal area at the plot-level. We used two groups of climatic variables, namely mean annual temperature and actual evapotranspiration as surrogates of environmental energy, and annual precipitation, precipitation seasonality, and water availability as surrogates of water availability. We found that AGB is more closely related to water availability variables than to energy variables. In northwest South America, water availability influences carbon stocks principally by determining stand structure, i.e. basal area. When water deficits increase in tropical forests we can expect negative impact on biomass and hence carbon storage.

## Introduction

Understanding and predicting the likely response of ecosystems to climate change are crucial challenges for ecology and for conservation biology [1]. A key ecosystem service and one of the most studied ecosystem characteristics in forests is the storage of carbon in trees. Knowledge of the distribution of above-ground biomass (AGB) is an essential basis for forest conservation strategies and programs, including the reduction of emissions by degradation and deforestation (REDD) [2,3], to be successful.

The challenge of understanding forest biomass is particularly important in tropical forests, where there are about 460 billion tons of carbon in their biomass and soil, equivalent to more than half the total atmospheric stock [4]. In addition, in terms of carbon fluxes tropical forests process 40 billion tonnes of carbon annually [5]. Deforestation and other anthropic processes significantly impact both stocks and fluxes [2], and recent droughts and climate trends are already impacting tropical biomass in Amazonia and elsewhere (e.g. [6]). There are several different approaches to understanding the distribution of tropical forest biomass. One is based on comparison of remote sensing data with stand variables (wood density, basal area and stem density), allowing AGB estimations [7]. This kind of correlative studies provide spatially-explicit and verifiable estimates of AGB and may allow for extensive assessments of carbon stocks [3]. This approach has great value for mapping carbon and evaluating risks from land-use change and potential benefits from policy interventions (e.g. [8]). Alternatively, environmentally based models can be developed to test and quantify potential ecological mechanisms controlling AGB and so have predictive properties, although the prediction accuracy for any given locality is likely to be low because of the multiple factors involved.

Biomass is the emergent outcome of many complex processes, acting at different temporal and spatial scales. From a reductionist point of view, however, it is determined by the balance between biomass inputs (i.e., growth) and outputs (mortality). We can expect therefore that conditions that favor high growth rates, such as abundant water supply, warmth, and nutrient-rich soils will tend to correlate with high biomass. Environments with high mortality risks–whether via wind extremes, extremes of temperature, or drought, will tend to support lower biomass. Water supply and temperature have multiple impacts on both growth and mortality processes, and so are likely to exert major control on AGB. This expectation is reinforced by the global pattern of covariation of ecosystem carbon turnover times with both precipitation and climate [9]. Within the tropics, different climatic variables have been found to covary with AGB in diverse regions around the world. For example, precipitation in the drier quarter is positively correlated with AGB in Amazonian forests [10]. In contrast, AGB is weakly related to climate across a latitudinal gradient in the Neotropics [11]. Models developed for broad climatic and geographic scales are not easily applied to finer scales and vice versa. Nonetheless, Stegen et al. [11] concluded that water availability does have an important effect on aboveground biomass, mainly via limiting the size of larger trees. Also, Baraloto et al. [12] found that AGB is locally more related to stand variables than to climate in Amazon forests. In turn, stand variables are related to the variation in soil water availability, which is determined by topography [13]. This suggests that climate effects on AGB in the tropics may vary regionally, and may be scale-dependent.

Other stand properties that are related to climatic factors could help explain climate impacts on biomass [14]. Notably, recent studies have shown the importance of large trees and their sensitivity to water availability as drivers of AGB. Stegen et al. [11] showed that the very largest tree in a stand are limited by water availability across forests in the Americas because the maximum potential stand AGB is climatically-controlled. Such a relationship appears to have a mechanistic basis in terms of water relations. Thus, for example, Phillips et al. [15] found that drought influences tree mortality across the world’s tropical forests and that the impact of water deficits on mortality rates is greatest for large trees. In addition, Nepstad et al. [16] based on induced drought experiments in Amazonian rainforests, showed that the mortality rates of large trees (> 30 cm of DBH) increased by 4.5 times on dry conditions, and are higher than mortality rates for small trees.

Recent studies have shown the risk of embolism increased in large trees, with hydraulic failure during dry periods linked to mortality (e.g., [17]). This mechanism should exert climatic control on stand AGB by impacting especially the physiology of larger trees and thus acting as a filter on tree size. The demonstrated global convergence in forest vulnerability to drought [18] is consistent with this, with safety margins against hydraulic failure being largely independent of precipitation, and mediated in part by changes in species composition and size (hence, AGB) along precipitation gradients.

Further, temperature is suggested to influence biomass because photosynthetic activity is temperature dependent. Clark et al. [19] showed that tree growth rates decreased in warmth years between 1984 and 2000, possibly due to photosynthetic reductions and respiration increments driven by the effect of maximum daily temperature. Also, Doughty & Goulden [20] showed a negative relationship between CO_2_ exchange and temperature, with a 3°C rise in environmental temperature causing a 35% decrease of gas exchange at the forest level. Other experiments found that the relationship between temperature and photosynthetic activity have a wider range than expected across plants species [21]. Thus, we can expect a hump-shaped relationship between biomass and temperature based on the temperature-dependence photosynthetic activity or a low plateau relationship, if high temperature does not reduce photosynthetic activity at the community level through wide species tolerances.

Important variation in tropical ecosystem properties at large spatial scales is associated with tens and hundreds of millions of years of evolutionary history, independent of climate. For example, the forests of the Pre-Cambrian Guyana Shield have up to 50% greater biomass density than forests growing in similar climate conditions on Neogene sediments in south-western Amazonia [22]. The lowland rainforests of Borneo are half as productive as climatically and edaphically matched equivalent forests in north-western Amazonia, a difference apparently driven by the preeminence of a single hyper-successful family, Dipterocarpaceae, in Southeast Asian forests [23]. Thus, analysis at cross-continental or multi-continental scales [11,15] might obscure true climatic impacts on tropical AGB due to geological and/or deep phylogenetic controls. To better identify and isolate the climate factors controlling biomass, it is therefore important to analyze tropical biomass variation at smaller scales, where such differences are proportionately less important. One approach is to analyze elevation transects along large mountain ranges (e.g. [24]). An alternative approach, taken here, is to analyze ecological variation within a few parts of the tropics where extremely long dry to wet and cold to hot climate gradients occur within relatively short distances, affording a rich set of contrasts and replication. Probably the single most climatically complex region of the world exists in northwestern South America. For example, three mountain ranges run from south to northeast, dividing lowlands and reaching an altitude of 6000 m (some peaks have permanent snow). The inter-Andean Magdalena and Cauca valleys, where dry forests are the predominant natural habitat, dissect these mountain ranges. In addition, two very wet forests (Chocó Biogeographic region and the Amazon basin) limit the Andean mountains.

Our main objectives are to i) quantify and (ii) describe the patterns of forest AGB stocks across major tropical climatic gradients and (iii) to understand the effects of water availability and environmental energy on stand variables, and indirectly on AGB, in Northwestern South America. In particular, we want to answer the following questions: i) Are stand variables (wood density, basal area and stem density) related to AGB along broad climatic gradients of temperature and moisture? ii) What is the shape of the relationship between stand variables and climate? iii) Is climatic variation related to AGB? Such answers will help improving our knowledge on tropical forest stocks and contribute to understand the likely effects of climate change on ecosystem functioning.

## Materials and methods

### Plot sampling and aboveground biomass estimation

**Fig 1:**
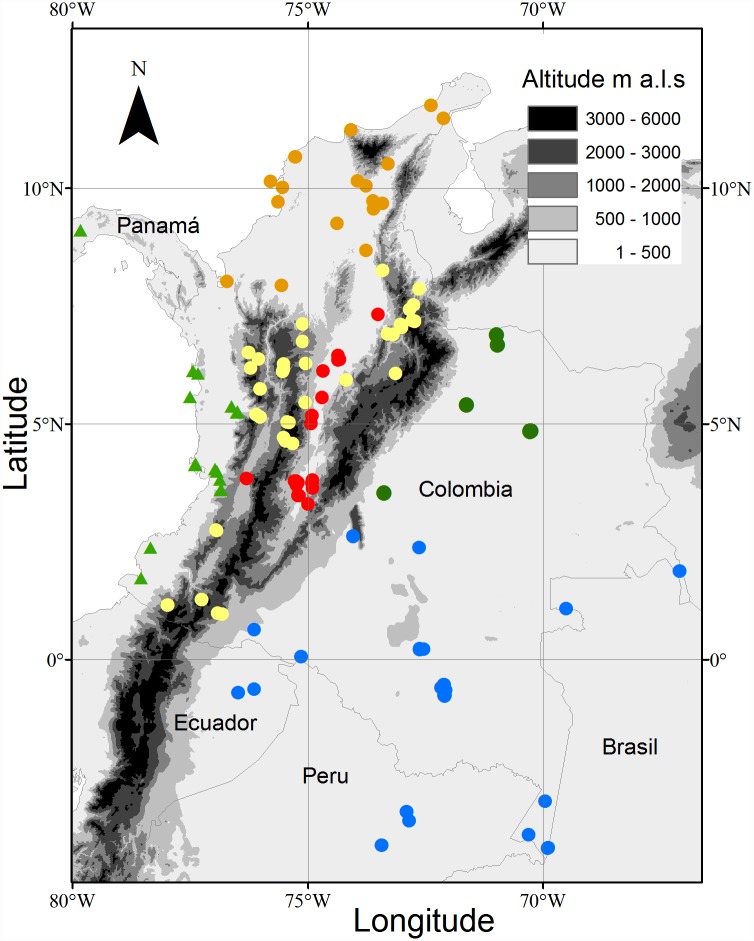
Distribution of plots used to estimate aboveground biomass in the studied Northwest South American region (Colombia, Brazil, Peru and Ecuador). Color of the symbols represent the forest types in the region: Blue for forest plots in Amazonia; yellow for forest plots in the Andean uplands; red for plots in the inter-andean valleys; Orange for the Caribbean plots; Green for the Orinoco region and the green triangles for the forest plot in the Choco region. The grey scale is displayed to denote altitude (m. a.s.l).

**Fig 2:**
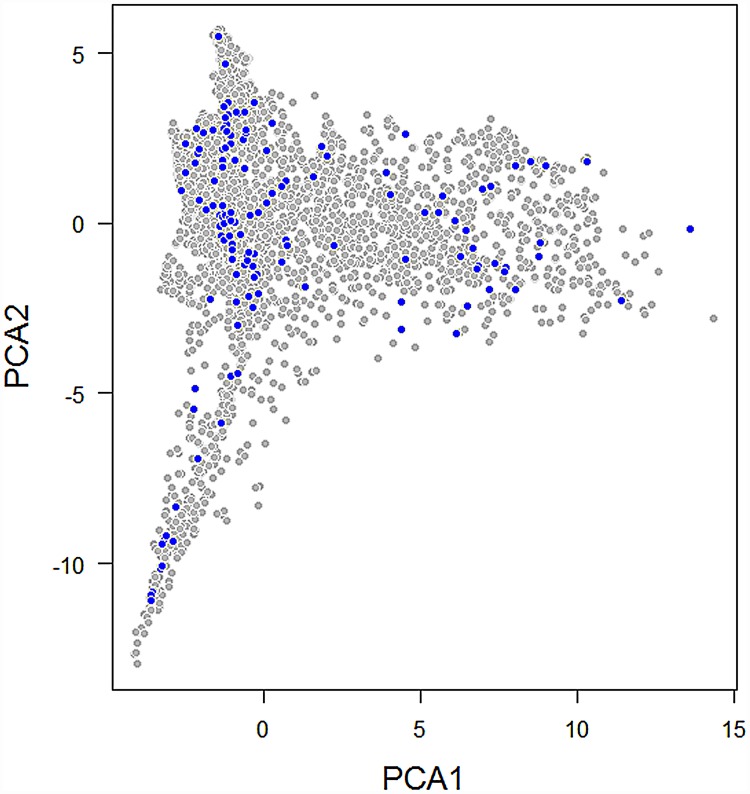
Climatic space represented for each vegetation plot used in this analysis. The climatic space is shown as principal components analysis to reduce climatic variables used. The first axis represents temperature variability and second axis represents precipitation variability. Gray points represent the climatic space availability across Northwest South America. Blue points represent actual climatic conditions of each of the vegetation plots sampled.

The sampled region (northwest South America) includes some of the wettest, driest, hottest, and coldest tropical forests on Earth. Within the region there is a large variety of forest types according to climatic and biogeographic regions: Amazon forests (moist forests), Andean montane forests (> 1000 masl), Chocó forests (pluvial forests), Caribbean forests (dry forests), Orinoquia forest (continuous and riparian forests) and, finally, inter Andean valley forests (a dry to moist lowland forest gradient along Magdalena and Cauca rivers). We gathered data from 156 plots in old-growth forest across the region at elevations ranging between 0 to 3400 m (Fig 1) in the period 1989–2013. The vast majority of the plots were established by the authors, using standardized methods [25], some of the plot data has been already published in recent work from the coauthors [22,26]. The vegetation plots are located in private-owned land and some in National Parks, for which we obtained permits. In some cases, an agreement was signed between the researchers and the landowners. In any case, this research did not focus on endangered species. These plots represent very large climatic gradients, notably from < 10°C to almost 30°C mean annual temperature, and from <1,000 mm to almost 10,000 mm annual rainfall (Fig 2). Plot area ranges from 0.25 to 25 ha, although most have an area of 1 ha. We converted all data to 1 ha equivalent units prior to analysis. Diameter at breast height (DBH) was measured for all trees in each plot. We used the values of wood density for a total of ~55% individual species reported in the Wood Density Global Database [27]. For 45% additional species not found in this database, acknowledging the strong phylogenetic signal for this trait [28], we used the genus or family mean, depending on data availability. For the relatively few cases when neither family nor genus were reported in our database (14%), we used wood density averaged per plot [29].

For our initial, exploratory analyses two sets of allometric equations were used to calculate AGB for each individual tree. One set is widely used for tropical analyses Chave’s AGB equation [30]), which has been reported to overestimate biomass stocks in some localities; the other set is based only on Colombian forest tree allometries (Alvarez’s GB equation [31]). We summed AGB values of all individuals in each plot to calculate AGB per plot (Mg ha^-1^). Both sets of equations in fact produced similar stand-level results (R^2^ = 0.97; F = 1165; p < 0.001) (S1 Fig). We will show here the results based on Alvarez et al. [31] equations, because these are regionally adjusted and allow controlling for allometric variation along the elevational gradient. As for AGB, we also derived plot-level values for stem density (N_ind_; N ha^-1^), basal area (BA; m^2^ ha^-1^), and wood density average (WD; g cm^-3^). In addition, we gathered 34 1ha plot from published studies which report Chave’s plot AGB and plot stand variables [22]; we transformed these Chave’s AGB values to Alvarez’s plot AGB using a previously fitted linear model. Finally, we included 10 additional plots from Colombia estimating the biomass indirectly from information on N_ind_ and BA. For this purpose, we use a model calibrated with the 156 plots for which we have the tree-by-tree data: AGB Mg ha^-1^ = exp (1.048 * ln (BA) + 0.299 * ln (Nin)); R^2^ -adjusted = 0.92, F-ratio = 833.0, P < 0.0001). Overall, we used 200 plots in further analyses. Data from the plots included in this study is available in the Supporting Information (S1 File).

### Climatic data

Climatic variables such as annual mean temperature (AMT; °C), annual precipitation (AP; mm) and within year precipitation variability (PV %; Variation coefficient of the monthly precipitation along the year) were downloaded from the WorldClim Global Climate Data [32]. Actual evapotranspiration (AET; mm) and potential evapotranspiration (PET; mm) were extracted from the Geospatial Database CGIAR Consortium for Spatial Information [33]. To distinguish the two main climate effects hypothesized to lead to spatial variation in AGB, we split climatic predictors onto two groups of variables representing environmental energy and water availability. We chose the following variables: AMT and AET, as surrogates of environmental energy; and AP, PV and water availability (WA mm; AP minus PET), as surrogates of water availability. Correlations between pairs of variables was moderate to low (maximum r = 0.68).

### Statistical analyses

To answer our questions, we followed five analytical steps: (1) We correlated pairs of explanatory variables, including climatic and stand variables, to examine collinearity. (2) We standardized explanatory variables by fitting a mean equal to 0 and variance to 1 to directly compare the effects of all explanatory variables. (3) We used generalized linear squares (GLS) models to relate each of these variables to AGB (response variable), and used generalized nonlinear squares (GNLS) models to test possible non-linear relationships. The parameters of GNLS were assigned using the brute force method, which consists of a heuristic approach to estimate model parameters. (4) We controlled the effect of spatial data distribution on model results by including a spatial correlation matrix on the models. This procedure allows detecting differences in parameter estimation when spatial variation is considered. (5) We used the Akaike Information Criterion (AIC) to choose between resulting models, and a pseudo-R^2^ (calculated as: (*Null deviance*—*Residual deviance*)/ *Null deviance*) to show explained deviance [34]. These analyses were conducted using packages *pls* [35], *nlme* [36], *proto* [37], *MASS* [38] and *raster* [39] from the R environment for statistical computing [40].

We applied the above five steps to evaluate the relationship between: i) stand variables (wood density, basal area and stem density) and AGB, ii) stand variables and climate, and iii) AGB and climate.

## Results

### Amount of aboveground biomass and structural parameters

Northwest South American forests showed huge variation in their AGB and structure (Table 1). AGB ranged between 7.7 and 386.9 Mg ha^-1^ (mean = 194.4, SD = 87.4), basal area between 1.6 and 46.8 m^2^ ha^-1^ (mean = 23.1; SD = 8.2), stem density between 61 and 1,388 ha^-1^ (mean = 583; SD = 214.7), and basal-area-weighted wood density between 0.37 to 0.75 g cm^-3^ (mean = 0.59; SD = 0.05). The highest and lowest values of AGB and BA were found in the Amazon forests and the Dry Inter Andean Valley forests, respectively (Table 1).

**Table 1:** Mean and standard deviation of aboveground biomass (AGB) across geographic regions of Northwest South America.

Region	N	AGB (Mg ha^-1^)		BA (m^2^ ha^-1^)		Stem density (N ha^-1^)		WD_BA_ (g cm^3^)	
		Mean	SD	Mean	SD	Mean	SD	Mean	SD
Amazonia	52	259.7	51.8	26.9	4.5	658.5	135.9	0.63	0.04
Andean total	63	211.9	70.8	26.0	7.3	689.6	191.0	0.57	0.02
Andean (Quercus present)	19	229.9	85.8	28.3	8.9	798.6	258.2	0.59	0.03
Andean (Not Quercus present)	44	204.1	62.8	25.0	6.3	642.6	131.1	0.57	0.02
Inter Andean valleys (Dry)	16	44.5	20.0	8.9	4.5	297.3	151.9	0.60	0.06
Inter Andean valleys (Moist)	10	156.6	41.6	20.8	4.8	618.5	225.0	0.57	0.05
Caribbean	19	75.4	52.7	14.4	8.6	340.8	195.0	0.59	0.07
Choco	35	217.5	46.6	24.8	5.0	554.3	155.7	0.55	0.05
Orinoquia	5	138.7	43.3	16.9	3.3	402.0	64.9	0.53	0.03
	200	194.4	87.4	23.1	8.2	582.6	214.7	0.59	0.05

N = plot number per region; AGB Aboveground Biomass; BA = Basal area; WD_BA_ = Wood density weighted by basal area.

### Correlations & explanatory models

**Fig 3:**
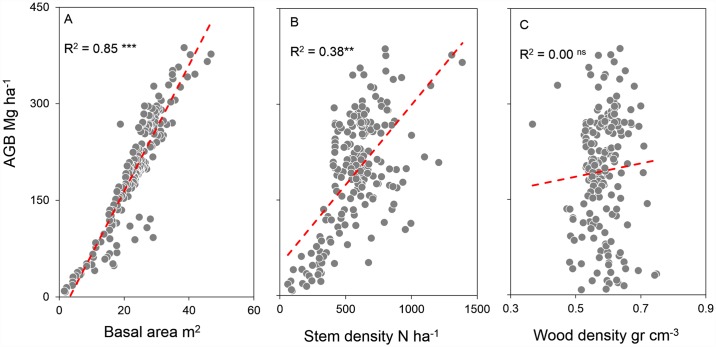
Plots of forest structure parameters and aboveground biomass. The Coefficient of determination is showed in each plot. *P<0.01; **P<0.001; ***P<0.000; ns: non-significant.

AGB was more strongly related with BA (R^2^ = 0.85, p < 0.001, Fig 3a) than with stem density (R^2^ = 0.38, p < 0.001, Fig 3b), whereas it was unrelated with wood density (R^2^ = 0.00; Fig 3c).

**Fig 4:**
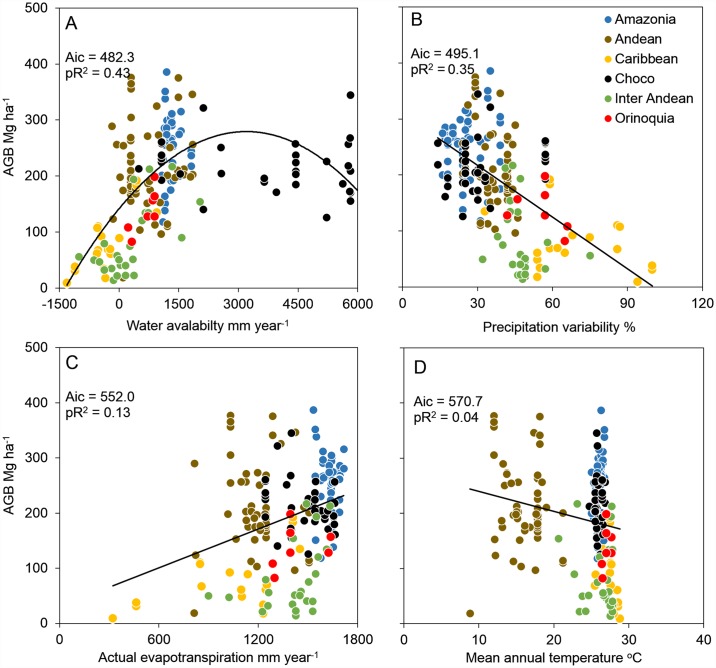
Relationship between Aboveground Biomass (AGB) and (a) Water Availability (WA), (b) Precipitation Variability (PV), Actual Evapotranspiration (AET) and (d) Annual Mean Temperature (AMT). Bioregions are shown with different colors. Solid lines represent the trend of relationships, based on the original data (without transformation), according to the best models (highest AIC scores) presented in Table 2; pR^2^ is a partial regression coefficient for each of the relationships.

AGB was significantly related to climate and stand structure variables and that it was more closely related to water availability variables (Fig 4a and 4b) than to energy variables (Fig 4c and 4d). Water availability (WA; annual precipitation minus potential evapotranspiration) was the single best determinant of AGB (Table 2). WA was better adjusted as a quadratic form, clearly suggesting a biomass maximum at intermediate levels of water availability; and lower biomass in sites with extreme precipitation. (Fig 4a). Additionally, precipitation variability (PV, i.e. the evenness of water supply through the year) was negatively related to AGB, suggesting that low rain variation promotes large biomass stocks in northwest South American forests (Fig 4b).

**Table 2:** Explanatory models of aboveground biomass.

MODELS	GLS (Only environment)							GLS (environment + space)						
	a	b	c	α	β	AIC	RSE	a	b	c	α	β	AIC	RSE
Water availability														
α (WA)^β^	-	-	-	1.920	0.307	527.8	0.896	-	-	-	1.883	0.324	513.2	0.914
a (WA)^2^ + b(WA)	-0.380	2.172	-	-	-	482.2	0.800	-0.370	2.137	-	-	-	473.0	0.818
b(WA) + c	-	0.312	1.634	-	-	559.3	0.953	-	0.340	-0.032	-	-	542.3	0.971
b(AP) + c	-	0.270	2.140	-	-	564.5	0.965	-	0.296	-0.033	-	-	547.6	0.985
b(PV) + c	-	-0.589	2.140	-	-	495.1	0.810	-	-0.594	-0.016	-	-	485.0	0.829
Environmental energy														
a (AMT)^2^ + b(AMT)	1.285	1.023	-	-	-	791.6	1.733	1.201	1.052	-	-	-	731.0	1.668
b(AMT) + c	-	-0.208	2.140	-	-	570.7	0.981	-	-0.233	-0.019	-	-	553.8	1.000
a (AET)^2^ + b(AET)	0.545	0.997	-	-	-	866.7	2.092	0.542	1.019	-	-	-	793.0	1.982
b(AET) + c	-	0.360	2.140	-	-	**552.0**	0.935	-	0.359	-0.024	-	-	**538.7**	0.958
Stand variables														
b(BA) + c	-	0.923	2.140	-	-	**200.3**	0.385	-	0.955	-0.018	-	-	**165.1**	0.404
b(WD_BA_) + c	-	0.058	2.140	-	-	578.8	1.001	-	0.072	-0.030	-	-	562.4	1.024
b(N_ind_) + c	-	0.623	2.140	-	-	482.1	0.784	-	0.614	-0.021	-	-	466.2	0.794

WA: Water availability; AP: Annual precipitation; PV: Precipitation variability; AMT: Annual mean temperature; AET: Actual evapotranspiration; BA = Basal area; N_ind_: Individual density; WD_AB_ = Wood density weighted by basal area. AIC = Akaike information criteria. RSE = Residual sum error. The best model based on AIC is bolded.

AGB was weakly related to actual evapotranspiration (AET) and annual mean temperature (AMT; Table 2). As these variables were highly correlated with elevation, this indicates little change in AGB across Andean mountains in Northwest South America. Finally, our assessment of spatial autocorrelation on AGB variation along climate gradients revealed that the model parameters were weakly affected by spatial variation (Table 2).

## Discussion

### Aboveground biomass variation across the study region

AGB values for the set of Amazon forests were within the range of previously reported values elsewhere in the Amazonian Basin (200–360 Mg ha^-1^; [10,41,42]). Similarly, our basal area estimates were within the range reported (25–35 m^2^ ha^-1^) in two large forest inventories [10,41]. In contrast, Choco and inter-Andean lowland tropical humid forest had lower values than expected (Table 1). Also, the studied Andean forests had higher values than previously reported at the pan-tropical scale [3], supporting the finding that mountain carbon stocks are still little known [43]. In addition, several Andean plots in our dataset have larger AGB than the maximum AGB value expected for Neotropical forests (350 Mg ha^-1^; [44]).

AGB pantropical maps [2,3], are widely used by governments when presenting proposals to reduce deforestation through economic compensations [45]; however, these maps can contain significant regional biases. Recently, Mitchard et al. [22] reported great differences between the AGB stocks reported in pantropical maps and the field data in the Amazon. In our study, in which we include a great number of field data, we also found great differences with the AGB estimations reported in pantropical maps (S2 Fig). First, AGB estimations extracted from these maps poorly predicts the observed AGB in the field, with an explained variation of only 24.0% for Saatchi et al [3] and 39.0% for Baccini et al [2]; Second, mean deviation, or error, of the AGB values predicted from the pantropical maps for each of our plots: Error = 100 * (AGB_predict_—GB_measured_) / AGB_measured_), is very high for both maps, with -45.8 ± 52.1% and 23.0 ± 109.5% for [3] and [2] respectively, showing low precision for these pantropical maps. This comparison confirms the findings by Mitchard et al. [22] and suggests that pantropical AGB maps should be revised to include the spatial variation in wood density [41] and the allometric relationship between tree diameter and height [46] to have more precise maps of carbon stocks in tropical forests.

### Water availability and forest structure

We found that water availability variables were highly related to AGB, rather than environmental energy variables (Table 2). This result is consistent with previous studies in the Neotropics [47] and African forests [48]. In northwest South America, water availability influences carbon stocks principally by determining stand structure, i.e. basal area. Also, this relationship is humped, which forests between 2500–3500 mm have higher values of AGB. For example, Choco and Amazon region have divergent values of AGB, although these may be considered structurally similar. Moreover, the influence of water supply on forest structure may be a pan-tropical phenomenon at different scales, for example Chave et al. [49] improved allometric equations including a similar water availability coefficient than ours. This suggests that it may be possible to improve the spatial accuracy of remote-sensing estimates of carbon stocks [22] by accounting for local water supply across the mapped surface. This is confirmed by Detto et al. [13] who demonstrated, in a mesic forest in Panama, that canopy height was highest in areas of positive convexity (valleys, depressions) close to draining channels, which seems like a response to greater water availability in the soil.

Water deficits in the shape of occasional or regular droughts are well-known to drive mortality, particularly of larger trees [11,15,16], and these mortality impacts may be limiting AGB in our forests too. The close dependence of AGB on BA (and hence on size of larger trees) is consistent with this mechanism. However, until sufficient long-term monitoring data are available it is unlikely to distinguish this from an alternative mechanism whereby extended dry seasons limit AGB simply by suppressing tree growth. Regardless of the exact mechanism, our results from a wide climatic gradient in a region with relatively little spatial extent underline the importance of water availability on AGB. If and when water deficits increase in tropical forests, we can expect negative impact on biomass and hence carbon storage [16]. However, future scenarios of precipitation remain rather poorly understood [50] and in recent years some Neotropical environments have become wetter (e.g. [51]). The future importance of water-limitation on AGB stocks in tropical forests thus remains unclear.

### Temperature and forest structure

We found a weak relationship between AGB and environmental energy variables, particularly with temperature. Several studies have shown that global temperature is an important determinant of the spatial distribution of biomass [52], due to its effect on the ecophysiological processes that control the net primary productivity rate (mainly photosynthesis and respiration). However, at the local or regional level, the tropical forests biomass is influenced by a large number of variables besides temperature and usually presents a high spatial variability. In the present study, the stronger effect of water availability than that of temperature hints that biomass output drivers could be more important than biomass input drivers shaping biomass stocks. Furthermore, temperature is correlated with elevation. This imply simultaneous changes in temperature, humidity, solar radiation, soils, species composition and historical factors that could affect biomass in concert [38,39,40] [53–55]. For example, Culmsee et al. [54] found higher AGB values in Southeast Asian montane forest plots dominated by Fagaceae species with high wood that contributed significantly to AGB. Similar biogeographic patterns may be occurring in Northern Andean forests that have landscapes dominated by temperate immigrants such as Fagaceae species (Table 1).

The ratio of height to diameter in tropical forests tends to decline with elevation and temperature (e.g. [31]), and so excluding such allometric variation by simply applying ‘universal’ allometric equations would lead to overestimating the biomass of Andean forests. Here, AGB was calculated using allometric equations specifically developed for Colombian forest types that implicitly include the known variation of tree height with elevation [31]. Additionally, we found that stem density was negatively related to temperature (Rp = -0.40; p < 0.001; i.e. positively related to elevation), similar to previous studies on tropical elevational transects [24,56]. Thus, other stand variables such as wood density could be important in driving this observation. However, wood density data of mountain species is scarce and requires more sampling effort [28], pointing to the challenge of AGB quantification on mountain ranges and the need of field work on these regions.

### Database and methodological limitations

Our study evidences that climatic variables, related to water availability are the most determinant of the spatial distribution patterns of primary forests of Northeastern South America. Nonetheless, there are some limitations, both in the data and the methods that could have affected our results. Regarding the data, the forest inventories do not include all the forest types and there are some missing points of the climatic gradient. For instance, the forests of the eastern Colombia (llanos), swamps, mangroves and highland forests (>3.000 m a.s.l.) are poorly represented; this fact may explain the high level of uncertainty of the predictive models for these forest types. Additionally, the distribution of the inventories is not random, mainly due to the intrinsic bias of ease of access in all tropical forest inventories [57], as well as the limited availability of forest remnants, such as the dry forests of the Caribbean. Regardless of these limitation, our data set includes forest plots of various sizes (0.25–1 ha) which is considered representative for the study of the structure of tropical forests [22].

Regarding the methods, the equations we used do not include height as a predictive variable of AGB, which could produce a bias in the estimations, given the fact that low precipitation and altitude can influence the positive allometry for tree diameter and height. However, the equations we used are calibrated for each forest type in the region and the specific allometry for diameter and height is intrinsically included in these biomass equations. Further similar analysis should include tree height as a variable to reduce uncertainty in the AGB estimations [46]. Another possible limitation of our methods is the resolution of the climatic data (1 km^2^) and the resolution of the forest plot data (1 ha or less). This lack of correspondence between climatic and forest data maybe ignoring the fine scale effects of the climatic heterogeneity on AGB [22,58]. Finally, some studies in the Amazon Basin have established that edaphic conditions maybe more important variables than climate determining forest structure and AGB stocks [14]. Future studies should include local edaphic variables.

## Conclusions

Water availability has an important role shaping spatial patterns of carbon stocks in northwestern South America across a huge climate gradient representative of the whole tropical forest zone, consistently with water deficits enhancing tree mortality and shifting size distribution of stands to favor small trees. AGB did not vary systematically with temperature, suggesting that temperature-mediated processes such as autotrophic respiration do not have a major impact on forest biomass in our study region. Increasing the understanding of how forest stand variables respond to climatic variability on spatial gradients could inform us about likely tropical biomass responses to climate change. By incorporating forest/climate relationships like those identified here it should be possible to improve calibration and accuracy of remote-sensing based maps of tropical forest carbon stocks.

## Supporting information

S1 File Biomass data used in the study. (XLSX)

S1 Fig Relationship between measured biomass with the Chave et al. (2005) and Alvarez et al. (2012) models, for 156 plots of Colombia. The lines represent the adjusted generalized linear model: ln (AGB *B1*) = a + b * ln (AGB *B2*) + *C*. Where *B1* = measured biomass in this study with Alvarez et al. (2012), *B2* = measured biomass with the Chave et al. (2005), and *C* = climatic categories of Chave et al. (2005), and *a*, *b* model coefficients. The model was significant (R^2^ = 0.97; F = 170; p < 0.0001). The resulting equations were: (Dry) *B1* = *exp* (0.124 + 0.924 * *ln* (*B2*)); (Moist) *B1* = *exp* (0.316 + 0.924 * *ln* (B2)); (Wet) *B1* = *exp* (0.342 + 0.924 * *ln* (*B2*)).
(TIF)

S2 Fig Relations between the biomass predicted by the pantropical maps of Baccini et al. (2012) and Saatchi et al. (2011), and the biomass measured in this study. The black solid line represents the adjusted linear model and the red dotted line the 1: 1 ratio between both values. For this analysis, we only use the 156 Colombian plots for which we have the data tree by tree. We have downloaded Saatchi map from https://www.arcgis.com/home/item.html?id=91d2f0e22e224366beedb4daef62179b and the Baccini map from http://www.whrc.org/mapping/pantropical/carbon_dataset.html.
(TIF)
