# Supplementary figures and images for: Forest biomass density across large climate gradients in northern South America is related to water availability but not with temperature
Source: PLoS One. 2017 Mar 16;12(3):e0171072. doi: 10.1371/journal.pone.0171072 (PMC5354365; doi:10.1371/journal.pone.0171072)

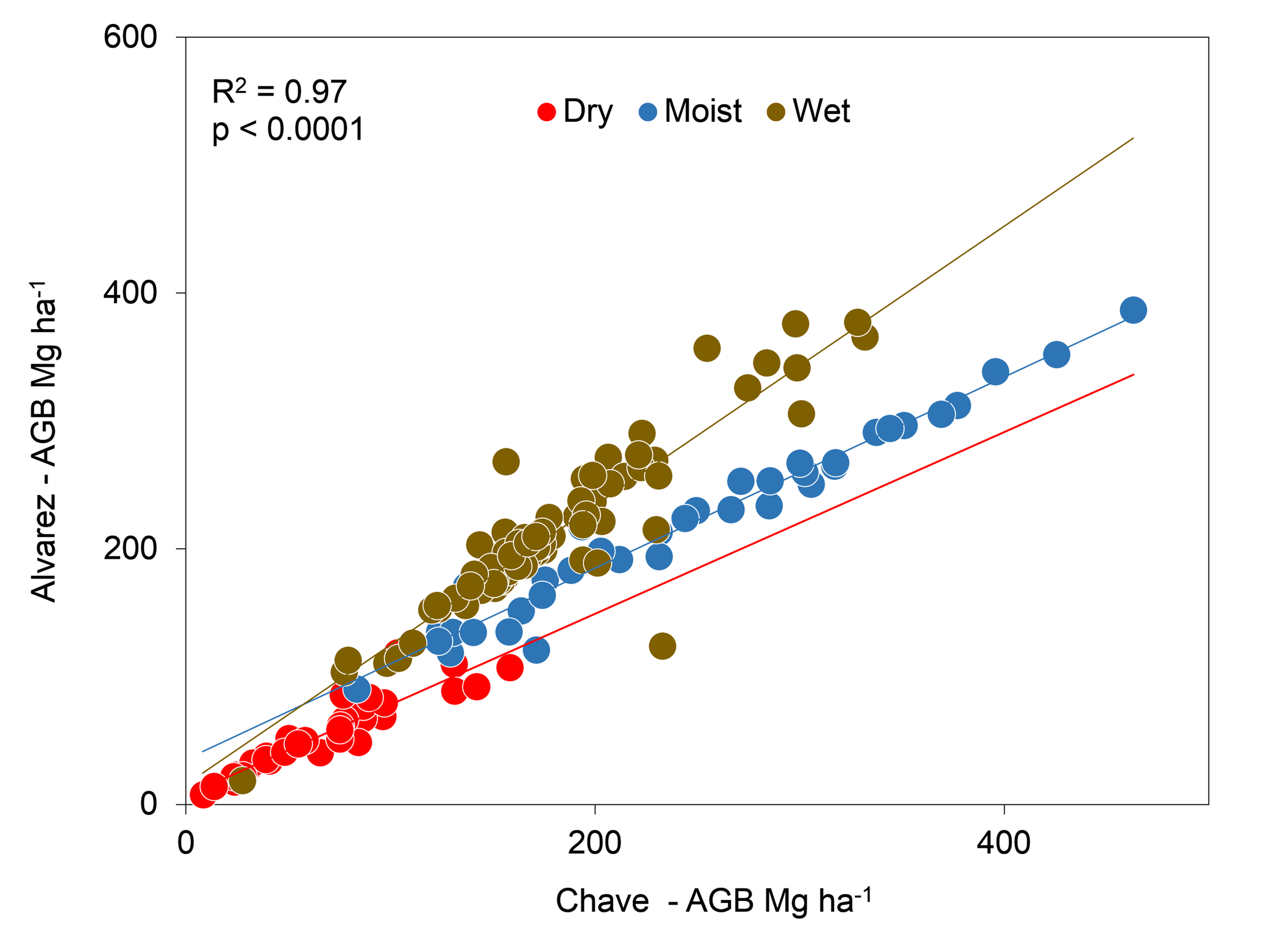

Supplement: S1 Fig — The lines represent the adjusted generalized linear model: ln (AGB B1) = a + b * ln (AGB B2) + C. Where B1 = measured biomass in this study with Alvarez et al. (2012), B2 = measured biomass with the Chave et al. (2005), and C = climatic categories of Chave et al. (2005), and a, b model coefficients. The model was significant (R2 = 0.97; F = 170; p < 0.0001). The resulting equations were: (Dry) B1 = exp (0.124 + 0.924 * ln (B2)); (Moist) B1 = exp (0.316 + 0.924 * ln (B2)); (Wet) B1 = exp (0.342 + 0.924 * ln (B2)). (TIF) [file pone.0171072.s002.tif]

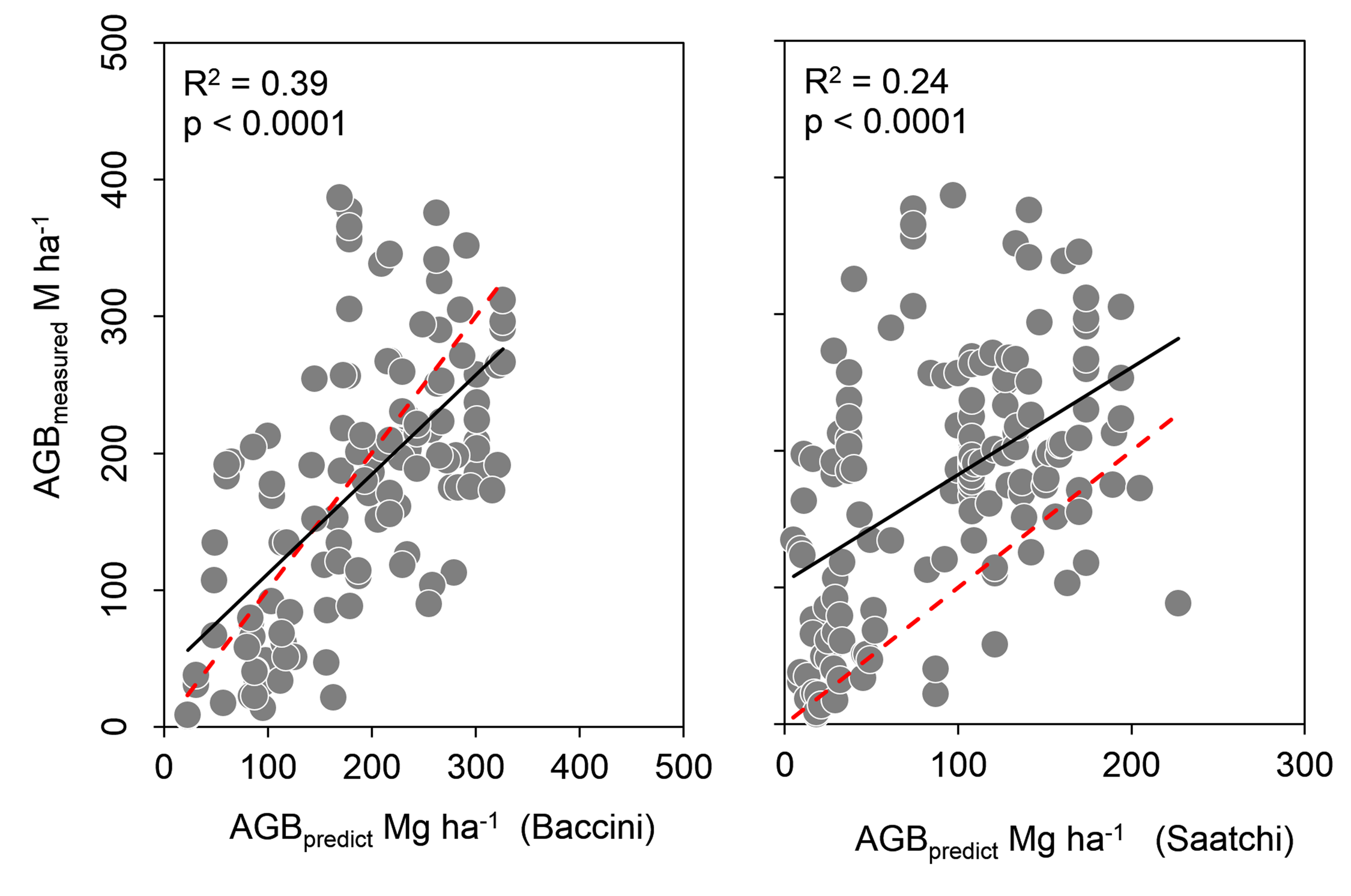

Supplement: S2 Fig — The black solid line represents the adjusted linear model and the red dotted line the 1: 1 ratio between both values. For this analysis, we only use the 156 Colombian plots for which we have the data tree by tree. We have downloaded Saatchi map from https://www.arcgis.com/home/item.html?id=91d2f0e22e224366beedb4daef62179b and the Baccini map from http://www.whrc.org/mapping/pantropical/carbon_dataset.html. (TIF) [file pone.0171072.s003.tif]
